# VitroBert: modeling DILI by pretraining BERT on in vitro data

**DOI:** 10.1186/s13321-025-01048-7

**Published:** 2025-08-06

**Authors:** Muhammad Arslan Masood, Anamya Ajjolli Nagaraja, Katia Belaid, Natalie Mesens, Hugo Ceulemans, Samuel Kaski, Dorota Herman, Markus Heinonen

**Affiliations:** 1 Johnson & Johnson, Beerse, Belgium; 2 Aalto University, Espoo, Finland; 3 ELLIS Institute, Espoo, Finland; 4 University of Manchester, Manchester, United Kingdom

**Keywords:** BERT, DILI, Toxicity, Molecular embeddings

## Abstract

Drug-induced liver injury (DILI) presents a significant challenge due to its complexity, small datasets, and severe class imbalance. While unsupervised pretraining is a common approach to learn molecular representations for downstream tasks, it often lacks insights into how molecules interact with biological systems. We therefore introduce VitroBERT, a bidirectional encoder representations from transformers (BERT) model pretrained on large-scale in vitro assay profiles to generate biologically informed molecular embeddings. When leveraged to predict in vivo DILI endpoints, these embeddings delivered up to a 29% improvement in biochemistry-related tasks and a 16% gain in histopathology endpoints compared to unsupervised pretraining (MolBERT). However, no significant improvement was observed in clinical tasks. Furthermore, to address the critical issue of class imbalance, we evaluated multiple loss functions-including BCE, weighted BCE, Focal loss, and weighted Focal loss-and identified weighted Focal loss as the most effective. Our findings demonstrate the potential of integrating biological context into molecular models and highlight the importance of selecting appropriate loss functions in improving model performance of highly imbalanced DILI-related tasks.

## Scientific contribution

We introduce VitroBERT-a general framework that extends traditional molecular pretraining by incorporating biological supervision. Our results demonstrate that molecular embeddings enriched with in vitro interactions outperform those based solely on chemical data. These findings reinforce the value of in vitro data in capturing biologically relevant information and highlight the model’s ability to leverage these features to model DILI risk.

## Introduction

Drug discovery is a complex and costly process, relying on a combination of in vitro, in vivo, and clinical studies to assess the safety and efficacy of potential therapeutic compounds [1]. In vitro experiments utilize isolated biological components-including microorganisms, cells, or biomolecules-extracted from their native physiological context. These studies are conducted under controlled laboratory conditions, allowing for precise manipulation and observation of specific biological processes [2]. Conversely, in vivo studies are performed within intact living systems, encompassing microorganisms, animals, humans, or whole plants, thereby preserving the complex interactions and regulatory mechanisms inherent to complete organisms.

In vitro models are employed to elucidate the pharmacokinetic properties of xenobiotics, specifically their absorption, distribution, metabolism, and excretion (ADME) profiles [3]. For instance, hepatic metabolism and potential toxicity can be assessed using primary hepatocytes or liver-derived cell lines such as HepaRG or HepG2 [4, 5]. These cellular systems enable the quantification of metabolic rates, identification of metabolites, and evaluation of cytotoxicity, providing crucial data on liver-specific drug interactions and potential hepatotoxicity [6]. In vitro studies offer a simplified, controlled environment to investigate biological systems at a molecular level. By isolating specific components such as hepatocytes or hepatic stellate cells, their roles in drug-induced liver injury (DILI) is investigated [7, 8]. These approaches facilitate detailed analysis of mechanisms underlying DILI, accelerates drug safety assessment, and reduces the reliance on animal models. Additionally, High-throughput screening can efficiently test drug candidates for potential hepatotoxicity, aiding in early drug development and reducing clinical trial failures due to DILI [9].

In vitro to in vivo extrapolation (IVIVE) is a critical challenge in translating research findings from controlled laboratory conditions to complex biological systems [10]. While in vitro studies offer detailed mechanistic insights, they often fail to accurately model outcomes in whole organisms. Factors such as drug delivery, metabolic processes, and complex tissue interactions can significantly influence drug efficacy and toxicity in vivo [11].

To bridge the gap between in vitro data and in vivo outcomes, particularly in the context of drug-induced liver injury (DILI), various modeling approaches have been developed. Traditionally, mathematical and statistical models such as physiologically based pharmacokinetic (PBPK) models have been employed to extrapolate in vitro findings to in vivo scenarios [12]. These models simulate the absorption, distribution, metabolism, and excretion (ADME) of drugs within living organisms using in vitro data from liver cells like hepatocytes [13]. By integrating physiological parameters such as liver blood flow, enzyme activity, and drug-binding properties, PBPK models can estimate drug concentrations and potential hepatotoxicity in the liver over time [14].

In recent years, many approaches have been proposed to reduce the gap between invitro-invivo extrapolation [15–17]. Robust DILI models have been proposed by combining molecular features, with other modalities such as transcriptomics [18], physicochemical properties [19, 20], and selected in vitro assays [21, 22].

Clinical DILI datasets are often small-scale, representing only a fraction of the vast chemical space, leading to models that struggle to generalize to unseen chemicals [23]. To address this limitation, researchers leverage representations derived from large amounts of unlabeled data [24]. These models transform data into a vectorized space, generating concise and well-structured representations that encompass broader chemical space [25]. To facilitate learning of underlying chemistry, various pretext tasks are carefully designed, including input translation between modalities [26, 27], input reconstruction [28, 29], and recovering masked or corrupted input [30].

In recent years, transformer-based models have been applied to molecular representation learning [31–34]. BERT (Bidirectional Encoder Representations from Transformers) is pre-trained on large text datasets using two objectives, defined by Devlin et al. [35] as the masked language model (MLM) and next sentence prediction (NSP) task. During pre-training, BERT learns bidirectional contextual embeddings for each token. By fine-tuning on task-specific labeled data, BERT adapts its learned representations to various downstream natural language processing tasks, achieving state-of-the-art performance. BERT can learn molecular representations by treating molecular structures represented as SMILES [36] as token sequences. Pre-training BERT on large molecular datasets with appropriate objectives, such as incorporating physicochemical properties or molecule relationships, enables it to learn robust chemical representations [37]. Fine-tuning the pre-trained BERT model on small task-specific labeled data can provide improved performance in various drug discovery applications [30].

**Fig. 1:**
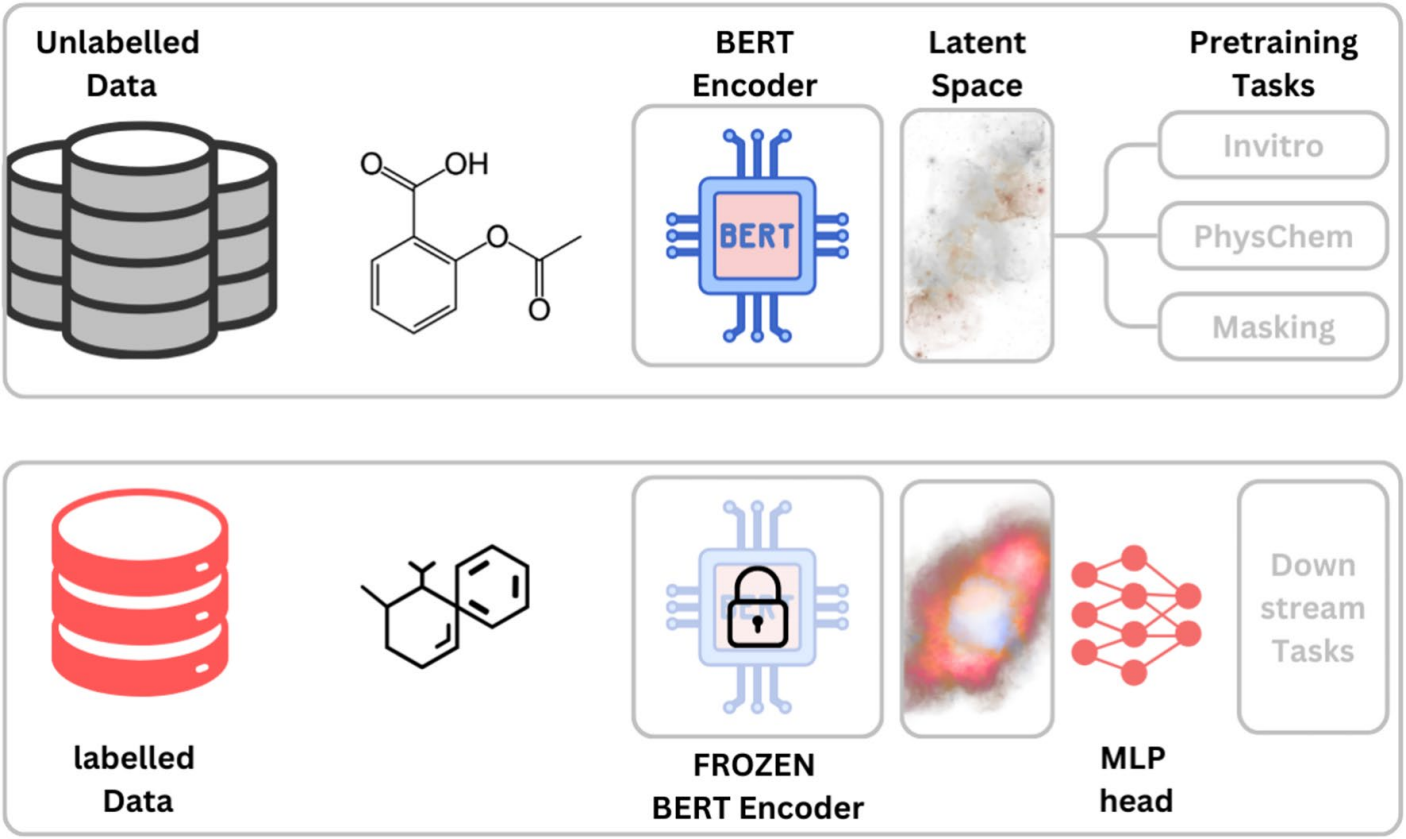
Overview of the VitroBERT framework: VitroBERT learns biologically informed molecular representations by jointly training on SMILES masking, physicochemical properties, and in vitro assays. This enables the model to capture both chemical and biological context. For downstream tasks, the pretrained BERT encoder is frozen, and an MLP head is trained on labeled data to estimate animal or clinical toxicity

These models excel at learning molecular context but often lack biological context, which is crucial for accurate toxicity modeling [38]. Integrating biological information, such as cellular phenotypes [38, 39] and -omics data [40], allows models to capture both chemical and biological responses to chemical exposure. To enable joint molecular representation learning from both chemical structure and corresponding biological annotations, we introduce VitroBERT-a general framework that extends traditional molecular pretraining by incorporating biological supervision. VitroBERT combines a shared BERT encoder with modular, task-specific heads for masking, physicochemical properties, and in vitro biological assays. The masking head captures SMILES grammar, the physicochemical head learns molecular intrinsic characteristic, and the in vitro head models biological interactions. This pretrained model yields biologically informed embeddings of downstream molecules, which are then used to train a lightweight multilayer perceptron (MLP) head for modeling animal and clinical toxicity as depicted in Fig. 1. We incorporated in vitro assays related to DILI-positive drug’s off-targets from the OFF-X database and ADME-related in-house assays to provide the relevant biological context. Our results demonstrate that embeddings enriched with in vitro interactions outperform those based solely on chemical data. To demonstrate the generalizability of the VitroBERT framework, we evaluate it on both publicly and proprietary datasets. While the public evaluation facilitates reproducibility, the in-house results highlight VitroBERT’s practical utility in real-world industrial settings.

## Materials and method

**Problem definition** We consider a modeling problem from molecules $\textbf{x}$ to binary toxicity profiles $\textbf{y}\in \{0,1\}^P$ of *P* endpoints from a dataset $D = \{(\textbf{x}_n, \textbf{y}_n)\}_{n=1}^N$ of size *N*. We assume a function $f(\textbf{x}; \theta ) \in [0, 1]^P$ that outputs separate probabilities for endpoints, and we use a shorthand $f_{np} = f(\textbf{x}_n; \theta )_p$. Molecules are represented by either strings by using SMILES notation, a binary vector $\textbf{x}\in \{0,1\}^n$ or a continuous vector $\textbf{x}\in \mathbb {R}^n$.

### Pretraining datasets

In this study, we used two pretraining in vitro datasets: a DILI-centric in-house dataset and the publicly available, curated ChEMBL20 dataset.

**J&J in vitro dataset** To extract DILI-centric in vitro dataset, we used OFF-X (the Off-Target Database), a comprehensive resource that compiles data on unintended interactions (off-target effects) of drugs and chemicals with proteins or receptors other than their primary targets, which can lead to side effects or toxicity. In our study, we utilized OFF-X to extract liver-related in vitro data. The OFF-X “drug-score” database lists associations of $\approx$7800 drugs with $\approx$11500 ADRs. Focusing on 172 liver-related clinical ADRs identified through MedDRA IDs, we identified $\approx$3000 drugs classified as liver-positive. Using the OFF-X “off-target” database, we found $\approx$1000 off-targets for these drugs. Upon querying J&J database to select assays that screened against these targets yielded approximately 7000 assays across 1.2 million compounds. These assays measure compound potency via pIC50, ranging from 4.3 to 6.0, corresponding to IC50 values of 50 $\mu$M to 1 $\mu$M. These assays are binarized by using several thresholds (with pIC50 > th), indicating compound activity against the target. We further enriched the data by including ADME-related assays-such as microsomal clearance, CYP450 inhibition, solubility equilibrium, permeability, and plasma protein binding. From this extensive dataset, we selected only assays with at least 100 active and inactive compounds, resulting in a final in vitro set comprising 1.26 million compounds across approximately 1200 tasks. Additionally, there is no overlap between pretraining and downstream molecules

**Public in vitro dataset** We also trained a separate model using only the publicly available ChEMBL20 dataset for in vitro pretraining. This manually curated dataset comprises $\approx$ 450,000 drug-like molecules annotated across 1310 binary classification tasks, including ADME, toxicity, physicochemical properties, binding, and functional activities [41]. All SMILES were processed using our standard pipeline (detailed in the Supplementary Information), and molecules appearing in downstream tasks were removed. Additionally, only targets with at least 10 positive and 10 negative samples were retained, resulting in a final dataset of $\approx$ 445,000 molecules and 1243 filtered targets.

### Downstream datasets

**J&J Preclinical dataset** This preclinical set integrates liver histopathology data from the TG-GATES dataset [42], which includes 138 compounds administered to rats under various concentrations and exposure conditions. We expanded this dataset to 412 molecules by incorporating J&J internal data. The histopathology labels were standardized to INHAND nomenclature for consistency [23]. The criteria used for mapping histopathology severity to numerical values is summarized in Table S1. Among the 55 liver endpoints, we selected 11 observed in at least 5 unique compounds: Pigmentation (P.D), Necrosis, Hypertrophy-Hyperplasia, Increased mitoses, Infiltration-Mononuclear, Cytoplasmic alteration (B.G.D), Single Cell Necrosis, Hypertrophy-Hepatocellular, Extramedullary Hematopoiesis, Cytoplasmic alteration (E.), and Vacuolation. Additionally, we included a global toxicity task, DILI_binary, which is positive if any of the classes show toxicity. Additionally, we incorporated 8 readouts from the TG-GATES biochemistry database: Alkaline Phosphatase (ALP), Aspartate Aminotransferase (AST), Alanine Aminotransferase (ALT), Gamma-Glutamyl Transferase (GTP), Total Cholesterol (TC), Triglycerides (TG), Total Bilirubin (TBIL), and Direct Bilirubin (DBIL). Binary histopathological labels were generated by pooling data across dose and time by using Equation S1, while biochemistry readouts were binarized using expert-defined thresholds given in Table S2; by using Equations S2-S4. The subset of this dataset is publicly available [23].

**Public Preclinical Dataset** To ensure reproducibility, we pretrained a separate model, VitroBERT-public, using the public in vitro dataset. We evaluated this model using the same 8 biochemistry readouts from TG-GATES data as in our internal dataset. These readouts were binarized with expert-defined thresholds, given in Table S2; by using Equations S2-S4. We extracted 138 compounds from the TG-GATES database. We excluded molecules with SMILES lengths exceeding 128, resulting in a final set of 133 compounds (see Supplementary Section: Preprocessing Pipeline), which were evaluated across these biochemistry endpoints.

**Clinical Endpoints** We used the publicly available SIDER dataset, containing 6,060 adverse drug reactions (ADRs) associated with 1,430 drugs; whose distribution is shown in Figure  S1 [43]. From this dataset, we identified 30 ADRs specifically related to drug-induced liver injury (DILI) within the system organ classes “Investigation” and “Hepatobiliary disorders” as highlighted in Figure S2. Detailed information on task selection and distribution is provided in the supplementary material. We excluded molecules with SMILES lengths exceeding 128, as well as those containing salts and metals, to ensure compatibility with pretrained MolBERT, resulting in a final set of 1217 clinical molecules (Table 1).

**Table 1:** Number of molecules and targets in each modality

Stage	Modality	Category	Targets	Unique Molecules
Pretraining	In vitro	Internal Assays	$\approx$ 1200	1.26M
	In vitro	Public Assays	1243	$\approx$ 445K
Downstream	Preclinical	Internal Pathologies	12	412
	Preclinical	Internal Biochemistry	8	412
	Preclinical	Public Biochemistry	8	133
	Clinical	SIDER	30	1217

“Preclinical” includes pathology and biochemistry tasks, while “clinical” refers to DILI-related tasks from the SIDER dataset. Two separate models were developed: one pretrained on internal in vitro data and evaluated on internal pathologies, biochemistry, and SIDER; the second pretrained on public in vitro data and evaluated on public biochemistry tasks

### Molecular representations

**ECFP** Extended-Connectivity Fingerprints (ECFP) is a method used in chem-informatics to represent molecular structures as binary fingerprints, capturing structural information by encoding the presence or absence of sub-structural features within a specified radius around each atom [44]. Through iterative traversal of the molecular structure, unique sub-structural fragments are identified and hashed into a fixed-length bit vector, generating a binary fingerprint where each bit indicates the presence or absence of a specific sub-structural fragment. We encoded each molecule into fix 1024 dimensional binary vector $\textbf{x}\in \{0,1\}^{1024}$ by using diameter 6 by using Algorithm S2.

**SMILES** The Simplified Molecular Input Line Entry System (SMILES) is a text-based notation that encodes chemical molecules as linear ASCII strings. For instance, aspirin, with the formula $C_9H_8O_4$, is represented by the SMILES string “CC(=O)OC1=CC=CC=C1C(=O)O”. We use canonical SMILES representations as input for the BERT model.

**Continuous representations** We used a BERT-based model to convert SMILES strings into dense, continuous high-dimensional vectors $\textbf{x}\in \mathbb {R}^{768}$. These continuous representations capture both local and global molecular contexts of the molecules. These embeddings serve as input for training task-specific MLP models to estimate endpoint-specific toxicity.

### Models

#### Baselines

We use Random Forest and multitask MLP as baselines, with both models utilizing ECFP fingerprints as input. We encoded each molecule into fix 1024 dimensional binary vector by using radius 6. Random Forest, an ensemble method combining decision trees, reduces over-fitting and provides robust, interpretable results while scaling efficiently for large datasets. We optimized task-specific hyperparameters using 5-fold cross-validation, as described in Algorithm S3. The hyperparameter search space is detailed in Table  S3.

The multitask MLP model consists of input, hidden, and output layers, initialized with ECFP features. We utilize dropout for regularization, batch normalization for training stability, and the rectified linear unit (ReLU) activation function as the default activation. Additionally, the network incorporates a skip connection, merging the input and output of the hidden layer, enhancing information flow. The final output layer produces logits, converted into probabilities via a sigmoid activation function (Equation S5, and Figure S4). 

We optimized the regularizer and loss function hyperparameters using 5-fold cross-validation. The search space and hyperparameters are detailed in Table S3.

### In vivo loss function

Given the nature of the problem, binary cross-entropy (BCE) is a suitable choice for training. However, toxicity datasets are often zero-inflated, with non-toxic (negative) samples significantly outnumbering toxic (positive) ones. Since BCE treats each observation equally, the model may become biased toward the dominant negative class. To address this imbalance, we introduce a weighted BCE, where the positive samples are up-weighted by the factor $w^{+}_p = \frac{N_{p-}}{N_{p+}}$, the inverse ratio of positives $N_{p+}$ to negatives $N_{p-}$ for each endpoint $p$. The weight $w^{+}_p$ can be further optimized by introducing a balancing parameter $\alpha$, determined through cross-validation:

1 $$\begin{aligned} w_{p}^+&= \alpha \frac{N_{p-}}{N_{p+}} + (1 - \alpha ) \end{aligned}$$

where $\alpha \in [0,1]$ controls the degree of positive balancing.

In cases of significant class imbalance, weighting alone may be inadequate, as it does not differentiate between easy (majority, non-toxic) and hard (minority, toxic) examples. Focal loss, originally developed for object detection, addresses this by incorporating a modulating factor that reduces the loss contribution from well-classified examples, thereby focusing on difficult, misclassified examples [45]. To assist focal loss further, we combine focal loss with positive weighting, resulting in our primary loss function: weighted focal loss

2 $$\begin{aligned} \mathcal {L}^{\textrm{w}}_{\textrm{FL}} = \sum _{n=1}^N \sum _{p=1}^P w^{+}_p \left( 1 - \sigma (f_{np})\right) ^\gamma y_{np} \log \sigma (f_{np}) + \sigma (f_{np})^\gamma (1-y_{np}) \log \left( 1- \sigma (f_{np})\right) \end{aligned}$$

Here, $y_{np} \in \{0,1\}$ is the ground truth binary label for sample *n* and endpoint *p*, $f_{np} \in \mathbb {R}$ is the corresponding raw model output, and $\sigma (\cdot )$ is the element-wise sigmoid function that maps model’s output to probability values in the range [0, 1]. This weighted focal loss (FL$^{w}$) reduces to focal loss (FL) when $\alpha = 0$, to weighted BCE (BCE$^{w}$) when $\gamma = 0$, and further to standard BCE when both $\alpha = 0$ and $\gamma = 0$.

#### MolBERT

The MolBERT model [37], an adaptation of BERT [35], features 12 attention heads, 12 layers, and a 768-dimensional hidden layer, with 85 million parameters. It is pretrained using two tasks: a masked token retrieval head with cross-entropy loss and a physicochemical property estimation head with mean squared error. The physicochemical properties, totaling 200, are computed using RDKit. The final loss is the arithmetic mean of these individual losses. This model is pretrained for 100 epochs using the Adam optimizer on 1.6 million SMILES from the GuacaMol benchmark dataset, which consists of compounds curated from ChEMBL [46]. We used this pretrained model to generate dense, continuous embeddings $\textbf{x}\in \mathbb {R}^{768}$ for preclinical and clinical SMILES. These embeddings served as input for a multitask MLP (Equation S5, and Figure S4). This strategy allowed us to leverage a significant volume of unlabeled data, and encapsulated the contextual information of larger chemical space. Further, we pre-trained MolBERT on J&J molecules using the same masking and physicochemical objectives. We refer to this domain-adapted model as “PhyschemBERT”.

#### VitroBERT

The VitroBERT model, based on the MolBERT encoder, features three specialized heads: PhysChemHead, InvitroHead, and MaskingHead. PhysChemHead and InvitroHead each include a single hidden layer, with PhysChemHead estimating physicochemical properties and InvitroHead focusing on in vitro tasks. The MaskingHead employs a linear layer, followed by a activation and normalization, then a single layer encoder to map hidden states to the vocabulary size. VitroBERT processes each batch twice: first with 15% of tokens randomly masked (corrupted sequence) and then without masking (clean sequence), generating embeddings $\textbf{h}_{\textrm{corr}}$ and $\textbf{h}_{\textrm{clean}}$. The MaskingHead uses $\textbf{h}_{\textrm{corr}}$ to identify the identity of masked tokens, while PhysChemHead and InvitroHead use $\textbf{h}_{\textrm{clean}}$ for modeling physicochemical properties and in vitro targets. Losses are calculated using Eqs. 2, 3, and 4. Similar to MolBERT, we used the trained VitroBERT models to generate dense, continuous embeddings $\textbf{x}\in \mathbb {R}^{768}$ for preclinical and clinical SMILES. These embeddings were then used as input features for a multitask MLP (Equation S5, and Figure S4) to model toxicity endpoints.

All VitroBERT models initialize their encoder weights from a pretrained MolBERT model, which was trained on 1.6 million SMILES strings for 100 epochs. This approach allows us to analyze the impact of different loss combinations on the encoder’s ability to capture and leverage molecular information. Table 2 presents different BERT variants along with their pretraining data.

**Table 2:** BERT variants with various heads configurations, and pretraining data sources

Encoder	Pretraining Data	Mask	Physchem	invitro
MolBERT	GuacaMol	✓	✓	✗
PhyschemBERT	Internal SMILES	✓	✓	✗
VitroBERT-internal	Internal in vitro	✓	✓	✓
VitroBERT-public	ChEMBL20	✓	✓	✓

Here, ✓indicates which head’s loss is used to train the embeddings

**Masking Loss** VitroBERT evaluates the performance of the masking task using the cross-entropy loss function, defined as:

3 $$\begin{aligned} L_{\text {masking}} = -\frac{1}{|\textbf{m}|} \sum _{i=1}^T m_i \cdot \log \left( \frac{\exp (\textbf{x}_{\text {pred}}[i, \textbf{x}_{\text {true}}[i]])}{\sum _{j=1}^{V} \exp (\textbf{x}_{\text {pred}}[i, j])} \right) \end{aligned}$$

where $\textbf{x}_{\text {pred}}[i, j]$ represents the model logits for token $i$ and vocabulary class $j$. $\textbf{x}_{\text {true}}[i]$ is the true token label for the $i$-th position that was masked. $m_i$ is a mask indicator where $m_i = 1$ if the $i$-th token was masked and $m_i = 0$ otherwise. $|\textbf{m}|$ denotes the number of masked positions, $V$ is the vocabulary size while $T$ represents sequence length.

**Physchem Loss** To evaluate the loss for physicochemical properties, we use the Mean Squared Error (MSE) loss defined as:

4 $$\begin{aligned} L_{\text {physchem}} = \frac{1}{n} \sum _{i=1}^{n} \Vert \mathbf {\hat{y}}_i - \textbf{y}_i \Vert ^2 \end{aligned}$$

where $\mathbf {\hat{y}}_i$ is the estimated vector of physicochemical properties for the $i$-th sample, $\textbf{y}_i$ is the true vector of physicochemical properties for the $i$-th sample, and $n$ is the number of samples. The notation $\Vert \cdot \Vert ^2$ denotes the squared Euclidean norm, which sums the squared differences between estimated and true values across all properties.

**In vitro Loss** For in vitro tasks, we employed the weighted focal loss with fixed parameters $\alpha = 1$ and $\gamma = 2$, as defined in the Eq. 2. No hyperparameter tuning was performed to optimize these parameters for the in vitro loss function.

### VitroBERT training and evaluation

#### Training

We developed two variants of VitroBERT: VitroBERT-internal, trained on J&J’s internal DILI-specific in vitro dataset, and VitroBERT-public, trained on public in vitro datasets as highlighted in Table 2. For VitroBERT-internal, the pretraining dataset was partitioned into training and validation sets using scaffold splitting, with the validation set comprising 100,000 unique SMILES. For VitroBERT-public, we utilized the public in vitro datasets (detailed in Section: 2.1) with $\approx$ 20,000 compounds reserved for validation. All VitroBERT variants were trained using Algorithm 1 (given in Supplementary), with the encoder initialized using pretrained MolBERT weights to expedite training. Each variant was trained for 10 epochs (equivalent to $\approx$350,000 training steps for VitroBERT-internal and $\approx$130,000 steps for VitroBERT-public). The progression of PhysChem loss, masked LM accuracy, and in vitro AUPR during the pretraining stage is shown in Figure S5. These plots highlight that VitroBERT-public continues to improve across all tasks as training progresses.

#### Evaluation

We evaluated VitroBERT-internal on internal preclinical pathologies, biochemistry endpoints, and DILI-related tasks from the SIDER dataset. For internal evaluation, we performed a 5-fold scaffold split, designating the fold with most diverse scaffolds as the test set to rigorously evaluate model generalization to novel and diverse set of chemical structures (Further details are in Supplementary section: Pre-Processing pipeline). Additionally, we assessed VitroBERT-public on biochemistry endpoints from the publicly available TG-GATES dataset comprising 138 molecules. During preprocessing, five molecules were dropped out, resulting in a final set of 133 molecules (see Supplementary Section: Preprocessing Pipeline). We used the predefined train-test folds from Chen et al. [47], employing both their random and molecular similarity-based splits, resulting 110 molecules in training and 23 molecules in test set. For both models, test sets remained isolated during development and were used exclusively for final model assessment and comparative analysis. The remaining data served for model training, with hyperparameter optimization conducted via 5-fold cross-validation. All performance metrics reported in Tables 3 and 4 represent model performance on these held-out test sets.

**Evaluation Metric** Given the imbalance between positive and negative instances, the ROC-AUC curve can be overly optimistic in highly imbalanced datasets [48, 49]. Therefore, we use Precision-Recall (PR) curves. The Area Under the Precision-Recall Curve (AUPR) quantifies a model’s performance by providing a single scalar value that summarizes its ability to accurately identify positive instances while maintaining precision. AUPR is particularly useful for evaluating models in datasets where the negative class significantly outweighs the positive class. However, for the sake of completeness, we report a whole range of metrics in supplementary material.

**Log-loss** To compute the loss of positive and negative instances for each task, we use the following equations:

5 $$\begin{aligned} \begin{aligned} \mathcal {L}^p_{\text {pos}}&= \frac{1}{N_{\text {pos}}}\sum _{n=1}^N \left( y_{np} \log \sigma (f_{np})\right) \\ \mathcal {L}^p_{\text {neg}}&= \frac{1}{N_{\text {neg}}}\sum _{n=1}^N \left( (1-y_{np}) \log (1- \sigma (f_{np}))\right) \end{aligned} \end{aligned}$$

## Results and discussions

**Fig. 2:**
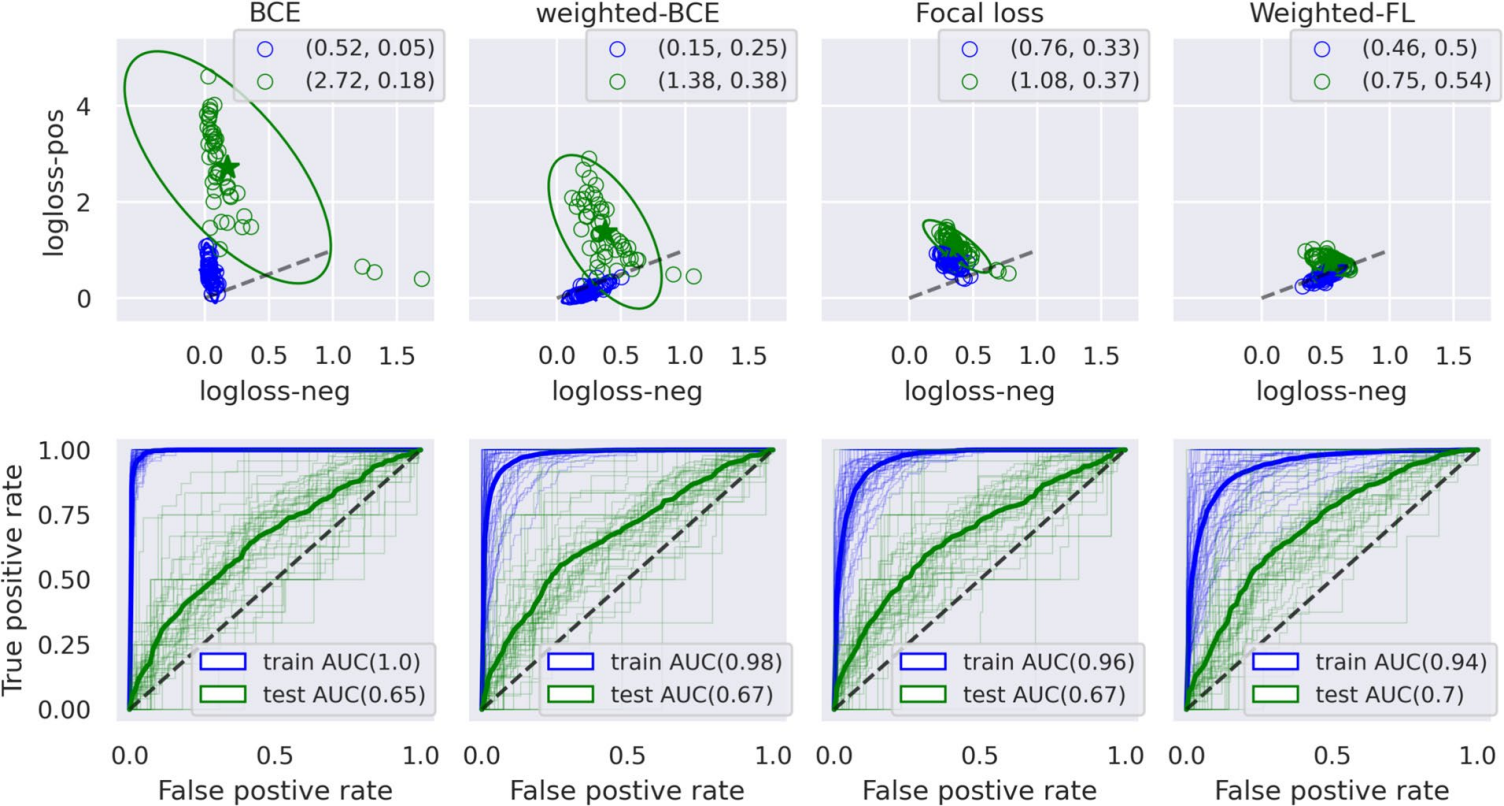
Effect of weighted losses (MolBERT-model). **Top row**: The wide gap in log-loss between positives and negatives indicates network bias towards negatives, mitigated by Weighted BCE and Focal Loss, with Weighted Focal Loss most effectively reducing log-loss for positives. **Bottom row**: ROC-AUC for train and test sets is shown, with light lines for task-wise values and a thick line for the mean; Weighted Focal Loss achieved the highest test ROC-AUC

To analyze the impact of different loss functions, we computed the log-loss for positive and negative instances generated from MolBERT, as shown in Fig. 2. Task-wise means, calculated using Eq. 5, are represented by small blobs with ellipses indicating the 95% confidence interval across all tasks. This visualization highlighted a network bias towards the majority class (negatives), resulting in elevated log-loss for positive instances, particularly with Binary Cross-Entropy (BCE). Transitioning to weighted Binary Cross-Entropy (BCE-W) prompted the model to equally prioritize both positives and negatives, decreasing log-loss for positives compared to BCE model. Moreover, Focal Loss naturally emphasizes on hard examples, in this case, positive (toxic) instances, lead to significantly lower log-loss for positives. Weighted Focal Loss further supported this by assigning additional weight to positive examples, consequently reducing the log-loss of positive instances even further. In our experience MolBERT model provided the highest ROC-AUC with weighted Focal loss as depicted in the bottom row of the Fig. 2. We opted for weighted focal loss as the default in vitro objective during VitroBERT pretraining, alongside masking and physicochemical losses (as detailed in Sect. VitroBERT).

**Table 3:** Performance of models across preclinical (internal biochemistry, pathology) and clinical (SIDER) tasks using different feature representations

Features	Predictive Head	In Vivo Loss	AUPR			
			Biochemistry	Pathology	Clinical	Combined
ECFP	RF	-	0.37 ± 0.031	0.21 ± 0.014	0.26 ± 0.011	0.27 ± 0.017
	MLP	FL	0.38 ± 0.068	0.30 ± 0.013	0.26 ± 0.016	0.28 ± 0.021
MolBERT	MLP	FL	0.36 ± 0.110	0.26 ± 0.024	**0.31** ± 0.021	0.31 ± 0.023
PhyschemBERT-internal		FL$^{w}$	0.45 ± 0.035	0.28 ± 0.023	0.29 ± 0.024	0.31 ± 0.022
VitroBERT-internal		FL$^{w}$	**0.51** ± 0.039	**0.31** ± 0.018	0.30 ± 0.026	**0.34** ± 0.022

MolBERT was trained on the GuacaMol dataset using masking and physicochemical heads. PhyschemBERT was trained on internal data using the same objectives as MolBERT, while VitroBERT additionally included an in vitro head trained on internal in vitro datasets. AUPR values are means across modality-specific tasks, with standard deviations from 5 runs using different random seeds. Best models per modality are shown in bold

In our experiments both Random Forest (RF) and and MLP models with ECFP features delivered comparable performance (Table 3). Incorporating pretrained MolBERT features resulted in a 16% improvement in clinical task outcomes as compared to ECFP features but fails to make a difference in preclinical task, indicating a lack of generalizable features for preclinical tasks among both ECFP and MolBERT, the Focal loss yielded the best results. In addition to AUPR, we also provide ROC-AUC of these model in Table S4.

In the next stage, we pre-trained MolBERT on J&J molecules using the same masking and physicochemical objectives, referred as PhyschemBERT. PhyschemBERT achieved a 20% improvement over MolBERT on biochemistry-related tasks. This gain is attributed to the alignment between the pretraining and downstream chemical spaces, despite having no overlapping compounds. Building on this, we introduced an in vitro head to MolBERT, alongside the masking and physicochemical heads, and jointly optimized the encoder using all three objectives. We refer to this model as VitroBERT. As shown in Table 3, VitroBERT yielded a 29% gain in preclinical biochemistry tasks, a 16% gain in preclinical pathology tasks, and an overall mean improvement of 9% across all tasks compared to MolBERT-without compromising clinical performance. This highlights the value of the in vitro head in capturing biological knowledge, as it models molecular interactions at the cellular level. These interactions are critical for understanding how toxicity manifests at the cellular stage, which provides a foundation for modeling broader toxicity outcomes in whole-animal systems.

**Fig. 3:**
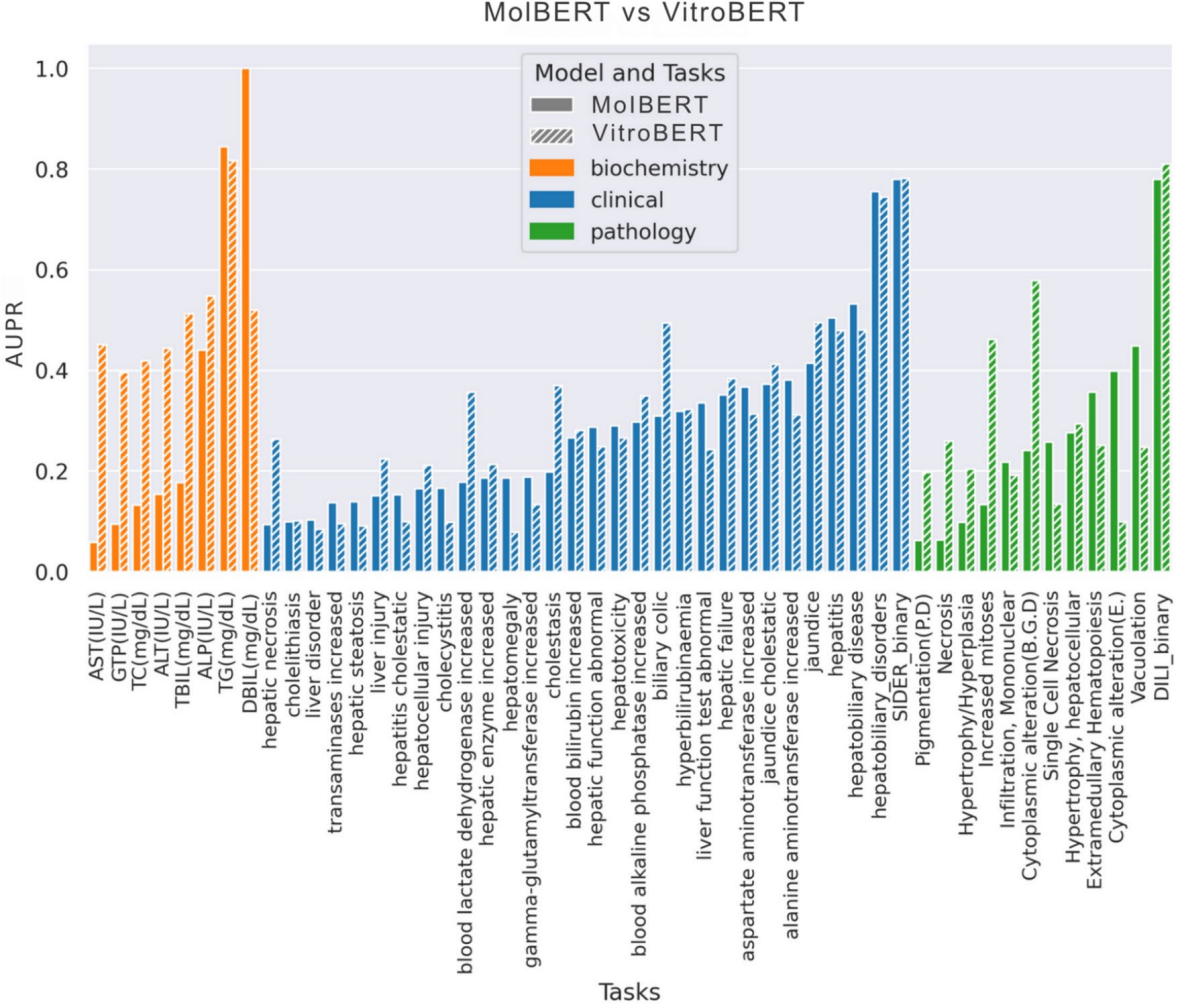
MolBERT vs. VitroBERT: Task-wise Comparison Bar color represents modality, while bar style differentiates between MolBERT and VitroBERT

**Fig. 4:**
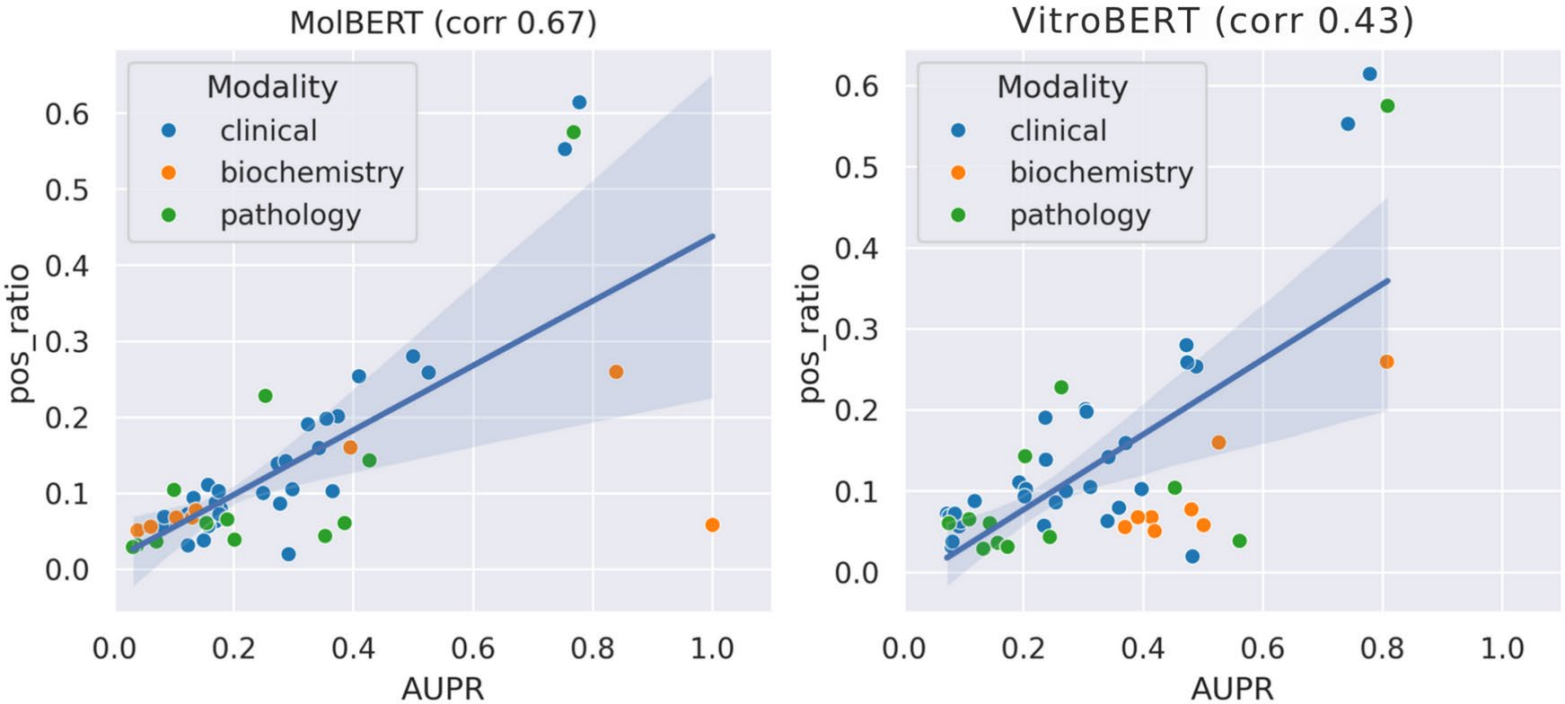
Effect of data imbalance on individual tasks in MolBERT and VitroBERT. VitroBERT shows lower sensitivity to class imbalance than MolBERT, as indicated by the lower Spearman correlation

A task-wise performance comparison between MolBERT and VitroBERT-internal is presented in Fig. 3. This analysis reveals that MolBERT’s performance is markedly skewed, excelling only in two biochemistry-related tasks-TG and DBIL-while significantly underperforming in other biochemistry tasks (AST, GTP, TC, ALT, TBIL, ALP). In contrast, VitroBERT demonstrates broader improvements, not only outperforming MolBERT across most biochemistry tasks but also achieving substantial gains in various preclinical pathology tasks such as Pigmentation, Necrosis, increased Mitoses, and Cytoplasmic alterations.

VitroBERT’s advantage is primarily evident in preclinical settings, with limited improvement in clinical DILI modeling. This may reflect the gap between in vitro data and the complex clinical manifestations of DILI, which involve multifactorial, host-specific responses [50]. Part of this stems from our modeling of liver-related adverse drug reactions (ADRs) from SIDER at the Preferred Term (PT) level of the MedDRA hierarchy-where signals are inherently weaker-whereas most prior methods operate at the broader System Organ Class (SOC) level [51]. Although VitroBERT shows only modest gains in estimating fine-grained DILI outcomes at the PT level, its performance at the coarser SOC-equivalent level is notably strong. To enable this comparison, we included a binary task, “SIDER_binary”, where any PT-level toxicity is aggregated into a single positive label. On this task, VitroBERT achieves $\approx$ 0.80 AUPR, indicating strong performance at a broader level of abstraction (as highlighted in Fig. 3). Furthermore, VitroBERT demonstrates substantial improvements in estimating other clinical ADRs such as Hepatic Necrosis, Liver injury, increased Blood Lactate, Cholestasis, and Biliary Colic, along with marginal gains in 13 additional tasks. These findings highlight the utility of in vitro pretraining.

Given the 50 tasks spanning three modalities, there is significant variability in class imbalance (the ratio of toxic to non-toxic samples) across tasks. To isolate the impact of task-specific class imbalance, we generated a scatter plot of AUPR versus positive ratio, with a regression line, as shown in Fig. 4. We also computed the Spearman correlation between AUPR and positive ratio, which indicates that MolBERT is more sensitive to class imbalance than VitroBERT. Notably, VitroBERT shifts the biochemistry tasks towards higher AUPR values, as highlighted by the orange points in the plot.

**Table:** VitroBERT-Public, pretrained on public in vitro datasets, was evaluated on 8 biochemistry-related endpoints from TG-GATES

Features	In Vivo Loss	Random Split			Similarity Split		
		AUPR	ROC-AUC	MCC	AUPR	ROC-AUC	MCC
ChemBERTa-77M-MLM [33]	FL$^{w}$	0.41 ± 0.017	0.42 ± 0.036	0.16 ± 0.021	0.37 ± 0.016	0.51 ± 0.024	0.26 ± 0.030
ChemBERTa-77M-MTR [33]	FL$^{w}$	0.48 ± 0.025	0.55 ± 0.013	0.24 ± 0.022	0.32 ± 0.028	0.47 ± 0.023	0.22 ± 0.018
MoLFormer-XL-10pct [52]	FL$^{w}$	0.46 ± 0.045	0.48 ± 0.029	0.23 ± 0.022	**0.40** ± 0.105	0.53 ± 0.085	0.28 ± 0.085
MolBERT [32]	FL$^{w}$	0.48 ± 0.015	0.62 ± 0.025	0.30 ± 0.024	0.37 ± 0.027	0.48 ± 0.022	0.25 ± 0.033
VitroBERT-Public	FL$^{w}$	**0.62** ± 0.066	**0.68** ± 0.031	**0.37** ± 0.037	**0.40** ± 0.037	**0.55** ± 0.043	**0.31** ± 0.027

Performance metrics represent means across all tasks, with standard deviations calculated from 5 independent training runs using different random seeds. Evaluations were conducted using both random and structural similarity splits. Results demonstrate VitroBERT’s superior performance over ChemBERTa, MolFormer, and MolBERT in both splitting strategies

We further validated our approach using public datasets by pretraining VitroBERT on publicly available in vitro data (ChEMBL20) and evaluating it on biochemistry-related tasks in the public TG-GATES dataset. Despite ChEMBL20 being less than half the size of the J&J in vitro dataset and offering no novel chemistry-since MolBERT had already been pretrained on similar structures-VitroBERT still showed significant performance gains across all metrics. Specifically, pretraining on public in vitro data led to a 23% improvement with random splits and a 7.5% improvement with similarity-based splits in AUPR as compared to MolBERT (as shown in Table 4). A comprehensive set of evaluation metrics is provided in Supplementary Table S5. We also compared full fine-tuning of VitroBERT with Low-Rank Adaptation (LoRA) across downstream tasks, with results summarized in the same table. The further details of these adaption methods are given in Supplementary, section “Hyperparameter search”.

We also benchmarked VitroBERT against several other pretrained models. These include ChemBERTa-MLM, pretrained on 77 million compounds using a masking objective, and ChemBERTa-MTR, pretrained on the same dataset using a regression objective to model 200 physicochemical properties computed with RDKit [33]. Additionally, we compared against MolFormer, which is pretrained on 1.1 billion compounds, although the publicly available version includes only 10% of this data ($\approx$ 100 million compounds) [52]. All of these models were pretrained solely on molecular data and did not incorporate any biological information. In contrast, VitroBERT learns biologically informed molecular representations and consistently outperforms all other models across all evaluation metrics-with one exception, where its AUPR is comparable to that of MolFormer as highlighted in Table 4. These findings reinforce the value of in vitro data in capturing biologically relevant information and highlight the model’s ability to leverage these features to model DILI risk.

All evaluations were conducted by extracting embeddings of downstream molecules using pretrained encoders (MolBERT, VitroBERT, ChemBERTa, and MolFormer), followed by training a MLP head optimized using the in vivo loss defined in Eq. 2. We performed 5-fold cross-validation using Algorithm S3, exploring the hyperparameter search space outlined in Table S3 to identify optimal settings for the MLP head, including the loss function parameters $\alpha$ and $\gamma$. On the J&J internal dataset, models using ECFP and MolBERT achieved the best performance with the standard Focal loss, whereas PhyschemBERT and VitroBERT achieved the highest AUPR when trained with the weighted Focal loss. On the public dataset, all models achieved optimal AUPR using the weighted Focal loss.

In conclusion, our experiments demonstrate that weighted focal loss outperforms BCE and weighted BCE for handling highly imbalanced datasets. VitroBERT, with in vitro pretraining, exhibits reduced sensitivity to class imbalance-a critical challenge in toxicity modeling-making it more robust and reliable for real-world datasets, where imbalanced classes are often prevalent.

While MolBERT, pretrained with masking and physicochemical heads, performs well in clinical tasks, it struggles in preclinical domains. This highlights the limitations of chemically-inspired pretraining, which does not adequately capture the complexity needed for effective generalization across diverse biological contexts. Domain adaptation through PhyschemBERT significantly enhances performance, as the in-house chemical space aligns more closely with downstream molecules, improving generalizability in preclinical settings. Additionally, incorporating biologically relevant information-such as pretraining on DILI-specific in vitro data and utilizing off-target information from DILI-positive drugs- enriches the model’s embeddings. This integration of biological context allows VitroBERT to better understand molecular interactions at the cellular level, which is crucial for modeling broader toxicity phenotypes observed in whole-animal systems.

Although the reported AUPR values may appear moderate, they exceed the performance of existing models for preclinical DILI modeling on noisy and imbalanced datasets such as TG-GATES. In early-stage drug development, even modest improvements can be valuable for deprioritizing potentially hepatotoxic compounds, thereby reducing downstream costs and risks. Overall, these findings demonstrate that combining domain-specific biological data with chemically-based pretraining provides a more comprehensive and effective approach for improving accuracy in toxicity tasks. VitroBERT’s ability to apply learned context from in vitro data to in vivo settings underscores the value of integrating both chemical and biological information.

## Additional file

Supplementary file 1 (pdf 1653 KB)

## Data Availability

The pretraining and downstream datasets can be downloaded from Figshare. The code and instructions to reproduce the results are available in the project’s GitHub repository: https://github.com/aidd-msca/Vitro-BERT.
