# Supplementary material for: VitroBert: modeling DILI by pretraining BERT on in vitro data
Source: J Cheminform. 2025 Aug 6;17:119. doi: 10.1186/s13321-025-01048-7 (PMC12326598; doi:10.1186/s13321-025-01048-7)
Supplement: Supplementary file 1 — (pdf 1653 KB) [file 13321_2025_1048_MOESM1_ESM.pdf]

VitroBERT - Modeling DILI by pretraining BERT on invitro data

Muhammad Arslan Masood,[*,†,‡] Anamya Ajjolli Nagaraja,[†] Katia Belaid,[†] Natalie Mesens,[†] Hugo Ceulemans,[†] Samuel Kaski,[¶,‡,§] Dorota Herman,[†] and Markus Heinonen[‡]

†*Johnson & Johnson*

‡*Aalto University, Finland*

¶*ELLIS Institute, Finland*

§*University of Manchester, United Kingdom*

E-mail: arslan.masood@aalto.fi

Material and Methods

Dataset

Histopathological data

In the preclinical setting, we model liver histopathology endpoints using the `TG-Gates` [1] dataset, a publicly available toxicological resource derived from `in-vivo` rat studies. The dataset includes a diverse set of compounds—many known to induce liver or kidney injury—administered at varying concentrations in both single- and repeated-dose settings.

The data contains gene expression, biochemistry, hematology and histopathology endpoints. The histopathological labels of this dataset has been re-annotated and transformed into `INHAND` labels [2]. In this study we utilized this updated version. In this setting, the toxic response of each molecule $\mathbf{x}_n$ is observed at multiple histopathological endpoints $p = 1, \ldots, P$ and over multiple animal replicates $k = 1, \ldots, K$, at various timepoints $t \in \{t_1, \ldots, t_8 \in \mathbb{R}\}$ and doses $d \in \{d_1, d_2, d_3 \in \mathbb{R}\}$. This dataset provides us a 5−dimensional observational matrix such that $\mathbf{y}_{i=(n,t,d,p)} \in \{y_i\}_{k=1}^{K}$, where $y_i \in \{s_0, s_1, \ldots, s_5\}$ is the severity of endpoint p of animal k at time t and dose d of molecule $\mathbf{x}_n$. The severities (s_j) are categorized into 6 levels as per table S1, which we map into range $s \in [0, 1]$. The animal replicates are not ordered and their counts K can vary.

Binary label assignment We pool the data over dose and time and only consider the 'nominal' toxicity y_{np}. The toxic instances associated with each phenotype (p) are counted $(\sum_k \mathbb{I}(y_{ndtpk} \neq s_0))$ at each drug-dose-time combination. A compound $(\mathbf{x}_n)$ is considered active in specific phenotype (p), if toxicity is observed in at least in two replicates $\sum_k \mathbb{I}(y_{ndtpk} \neq s_0) \geq 2$.

$$y_{np} = \begin{cases} 1, & \text{if } \exists \text{ a combination } (d,t) \text{ such that } \sum_k \mathbb{I}(y_{ndtpk} \neq s_0) \geq 2 \\ 0, & \text{otherwise} \end{cases} \tag{S1}$$

Table S1: The toxicity severity criteria

Severity	Proportion of liver affected	Grade	Quantification	Mapped value
NonToxic	No damage	0	-	0.0
Minimal	Very small amount	1	3–4 cells thick	0.2
Slight	Small amount	2	5–6 cells thick	0.4
Moderate	Medium amount	3	7–8 cells thick	0.6
Marked	Large amount	4	9–10 cells thick	0.8
Severe	Very large amount	5	> 10 cells thick	1.0

Biochemistry endpoints

To extend preclinical tasks further, we also considered including the selected readouts (ALP, AST, ALT, GTP, TC, TG, TBIL, DBIL) from TG-GATES biochemical dataset. In this setting, the multiple biochemicals $p = 1, \ldots, P$ are measured over multiple animal replicates $k = 1, \ldots, K$, for each molecule ($\mathbf{x}_n$) at various timepoints $t \in \{t_1, \ldots, t_8 \in \mathbb{R}\}$ and doses $d \in \{d_0, d_1, d_2, d_3 \in \mathbb{R}\}$. Here, d_0 is representing the control group. The outputs $y_{ntdpk} \in \mathbb{R}$ corresponds to concentration of p-marker in k-replicate at d-dose,t-time, for n-drug. We used expert defined thresholds (given in table S2) to convert continuous response values ($y_{\text{obs}} = y_{ntdpk} \mid d \notin \{0\}$) in binary labels $\mathbb{R} \to \{0, 1\}$ by comparing it with control group ($y_{\text{control}} = \frac{1}{K} \sum_{k=1}^{K} y_{ntdpk} \mid d \in \{0\}$).

$$y'_{ndtpk} = \begin{cases} 1, & \text{if } y_{\text{obs}} > \text{th} \times y_{\text{control}} \\ 0, & \text{otherwise} \end{cases} \tag{S2}$$

For TBIL and DBIL, we generated binary labels according to equations S3 and S4, respectively.

$$\text{TBIL\_label}(y_{\text{obs}}, y_{\text{control}}) = \begin{cases} 1, & \text{if } 0 \leq y_{\text{control}} < 0.1 \text{ and } y_{\text{obs}} > 0.1 \\ 1, & \text{if } 0.1 \leq y_{\text{control}} < 0.25 \text{ and } y_{\text{obs}} \geq 0.35 \\ 1, & \text{if } y_{\text{control}} \geq 0.25 \text{ and } y_{\text{obs}} \geq 0.5 \\ 0, & \text{otherwise} \end{cases} \tag{S3}$$

$$\text{DBIL\_label}(y_{\text{obs}}, y_{\text{control}}) = \begin{cases} 1, & \text{if } y_{\text{control}} \leq 0.1 \text{ and } y \geq 0.15 \\ 1, & \text{if } y_{\text{control}} > 0.1 \text{ and } y \geq 0.3 \\ 0, & \text{otherwise} \end{cases} \tag{S4}$$

Further, We pool the data over dose and time and compute labels for 'nominal' toxicity y_{np}, by using equation S2, similar procedure we performed for pathological data.

Table S2: Thresholds of Biochemical readouts to generate label

**Description**	**Thresholds**
Alkaline Phosphatase (ALP)	1.5
Aspartate Aminotransferase (AST)	2
Alanine Aminotransferase (ALT)	2
Gamma-Glutamyl Transferase (GTP)	3
Total Cholesterol (TC)	1.5
Triglycerides (TG)	3
Total Bilirubin (TBIL)	dependent on $y_{control}$
Direct Bilirubin (DBIL)	dependent on $y_{control}$

Clinical dataset

We are using SIDER dataset that provides 6060 adverse drug reactions (ADRs) of 1430 drugs, and in-total 162363 drug-ADR pairs [3]. It provides frequency information for 39% of drug-ADR pairs, with 19% allowing comparison to placebo treatment frequencies.
In the clinical dataset, 95% of drugs have over 10 adverse drug reactions (ADRs), and around 17% of ADRs are associated with at least 25 drugs. The distribution of ADRs and drugs have been shown in figure S1. We excluded molecules with SMILES lengths exceeding 128, as well as those containing salts and metals, to ensure compatibility with pretrained MolBERT.

Clinical DILI ADR

MedDRA hierarchy MedDRA (Medical Dictionary for Regulatory Activities) is a standardized medical terminology used for the classification and coding of adverse events and drug-related effects. All adverse drug reactions (ADRs) in this study adhere to the standardized terminology provided by MedDRA. MedDRA employs a hierarchical pyramid system consisting of five levels, with the highest level being the system organ class (SOC), which

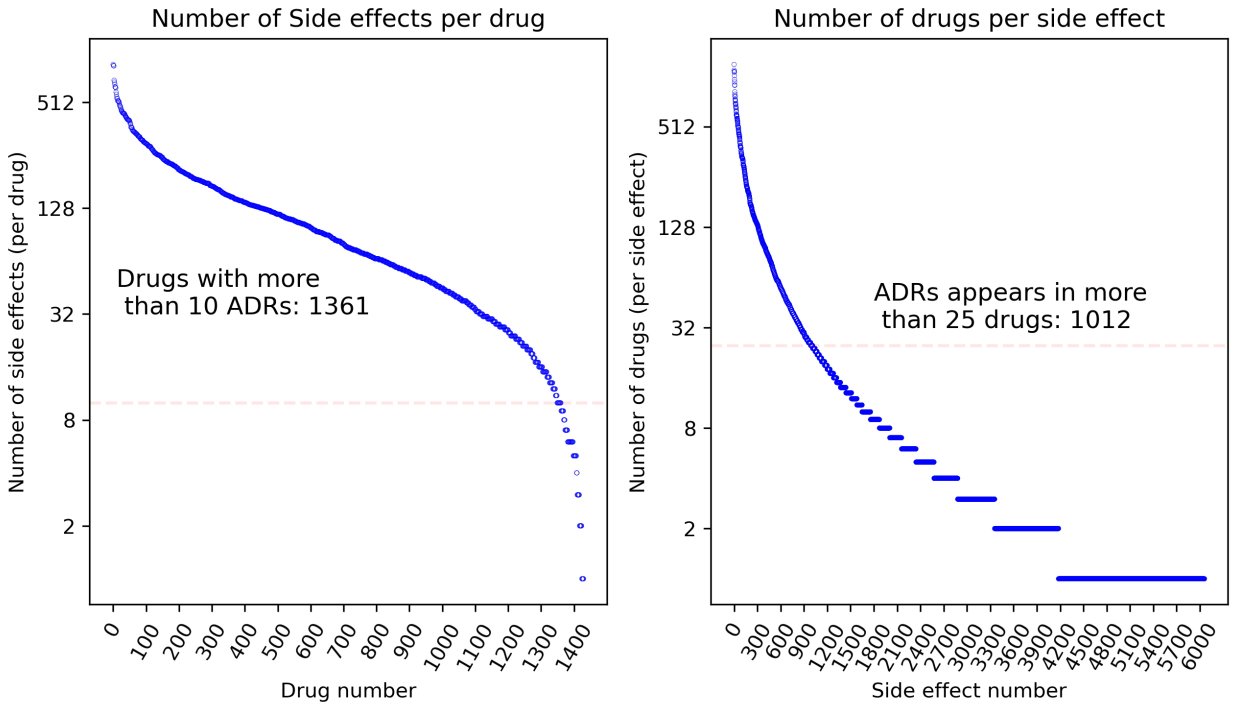

Figure S1: clinical: The distribution of 1430 drugs over 6060 adverse drug reactions (ADRs) and vice versa.

offers the broadest granularity. As we descend the hierarchy, the level of granularity becomes more specific. Our modeling efforts primarily focus on the fourth level of granularity, known as Preferred Terms (PTs), as they provide the most precise information regarding drug ADRs. The lowest level, Lowest Level Term (LLTs), mostly comprises synonyms of Preferred Terms, thus making PTs the most suitable choice.

Here is the description of MedDRA hierarchy:

1. System Organ Classes (SOC): 27

   The broadest level, grouping terms based on the affected organ system. For example, "Hepatobiliary disorders" is an SOC within the hierarchy.

2. High-Level Group Term: >330

   This level further categorizes the SOC into high-level group terms. For hepatobiliary disorders, examples of high-level group terms could be "Liver and biliary disorders".

3. High-Level Term (HLT): >1700

   HLTs provide more specific descriptions of the high-level group terms. An example within hepatobiliary disorders could be "Liver disorders NEC" (Not Elsewhere Classified)

4. Preferred Term (PT): >20000

   PTs are the most specific terms in the hierarchy and represent individual medical concepts. Examples within hepatobiliary disorders include "Hepatitis" and "Cholelithiasis".

5. Lowest Level Terms (LLTs): >70000

   These terms provide even more specific details, including synonyms and alternative wordings for PTs.

Hepatobiliary-related side effects The clinical dataset's adverse drug reactions (ADRs) are directly translated into MedDRA terms. The distribution of drugs and adverse drug reactions (ADRs) in each System Organ Class(SOC) is shown in figure S2. To ensure a comprehensive selection of adverse events associated with drug-induced liver injury, we reviewed the full list of adverse event terms from the MedDRA terminology. Initially, we selected terms directly associated with liver conditions, categorized under the "hepatobiliary disorders" SOC. Subsequently, we reviewed the "investigations" SOC for terms linked to laboratory tests indicative of liver dysfunction. This approach yielded 69 preferred terms under "hepatobiliary disorders" and 18 under "investigations." We refer to these combined ADRs as "HepSEs" (Hepatobiliary-related side effects). To optimize the search space for QSAR modeling, we focused on ADRs associated with at least 25 molecules, resulting in a final selection of 30 HepSEs.

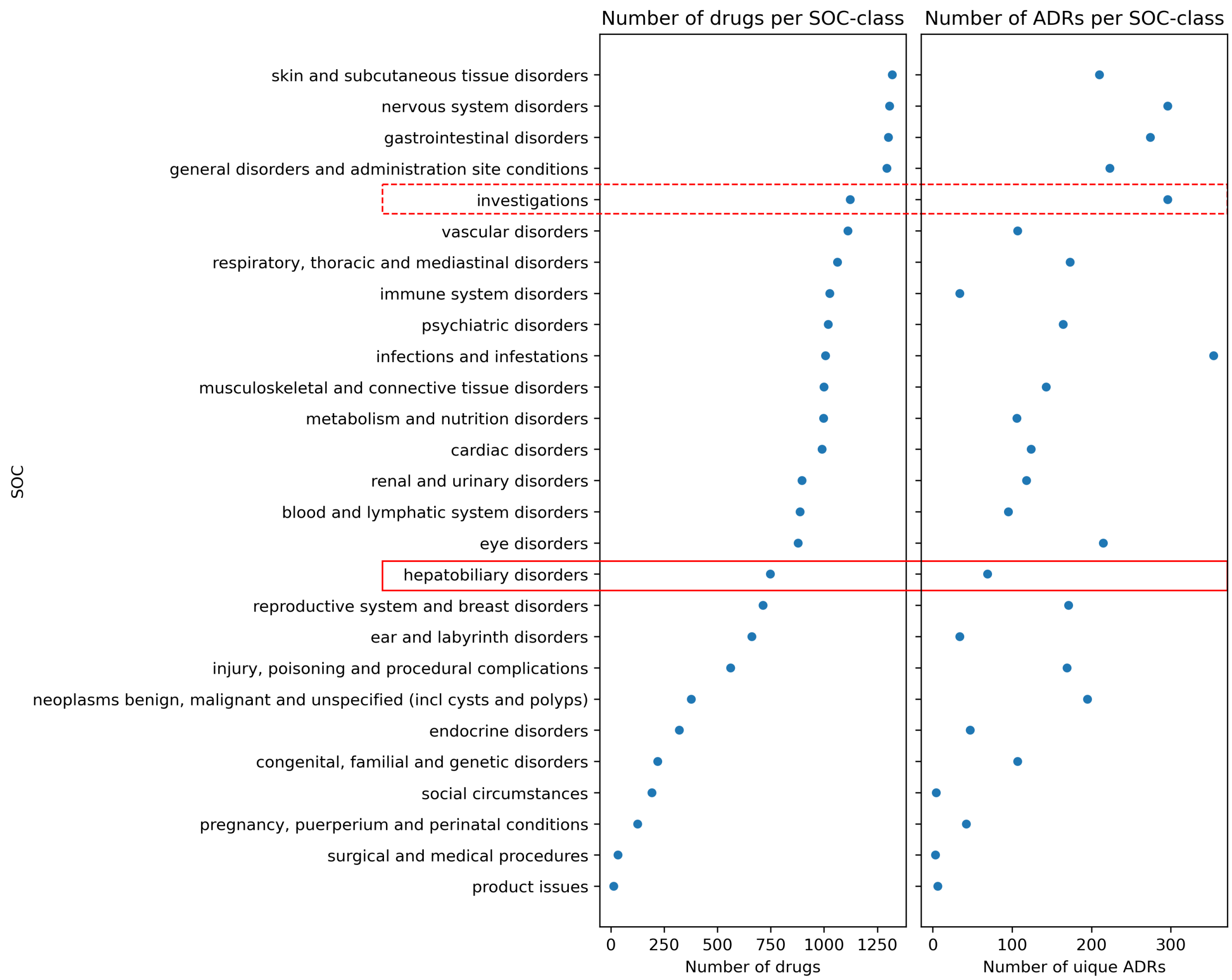

Figure S2: clinical: The distribution of drugs and adverse drug reactions (ADRs) in each System Organ Class (SOC)

Pre-Processing pipeline

To ensure uniformity, all SMILES were processed through an RDKit-based pipeline [4]. The SMILES standardization pipeline involves the removal of hydrogen atoms, dissociation of metal atoms, normalization of SMILES strings, reionization, elimination of salts, and canonicalization, yielding uniformly standardized molecular representations. We excluded molecules with SMILES lengths exceeding 128, as well as salts and metals, to ensure compatibility with pretrained MolBERT. This filtration results in the exclusion of 108 compounds from SIDER, and 5 molecules from public TG-GATES

Further, we computed the scaffolds based on the Bemis-Murcko scaffold representation [5], which identifies rings, linkers, atomic properties, hybridization and bond order. Employing the RDKit implementation, we identified 1035 unique scaffolds. For data partitioning, the StratifiedGroupKFold method from scikit-learn was employed [6], creating 5 folds. This method ensured the allocation of molecules from the same scaffolds to the same fold, thereby maximizing diversity across folds. Furthermore, stratification is performed based on "DILI_binary" task to ensure equal spread of preclinical toxic compounds across each fold. In the final stage, we identified a fold with the most diverse scaffolds and marked as "test set", comprising approximately 20% of the data, while the remaining folds were labeled as training data. The test set remains undisclosed and will solely be used for model evaluation and performance comparison. Hyperparameter selection will be executed through 5-fold cross-validation using StratifiedKFold on training set.

Models

Feature Extraction

We used Algorithm S2 to convert downstream SMILES into ECFP and BERT features. MLP heads were then trained for preclinical and clinical tasks by using these features. In this context, "BERT" serves as an umbrella term encompassing MolBERT and all VitroBERT variants.

In vivo head

Invivo head used for mapping ECFP and BERT features to preclinical and clinical toxicity modeling.

$$
\begin{aligned}
\mathbf{x}_0 &= \mathbf{x} \quad \texttt{BERT features or ECFP} \\
\mathbf{x}_\ell &= \text{Dropout}(\text{ReLU}(\text{BatchNorm}(W_\ell \mathbf{x}_0 + \mathbf{b}_\ell))) \\
\tilde{\mathbf{x}}_{\ell+1} &= \text{BatchNorm}(W_{\ell+1}\mathbf{x}_\ell + \mathbf{b}_{\ell+1}) \\
\mathbf{x}_{\ell+1} &= \text{Dropout}(\text{ReLU}(\mathbf{x}_\ell + \tilde{\mathbf{x}}_{\ell+1})) \\
x_{out} &= W_{\ell+2}\mathbf{x}_{\ell+1} + \mathbf{b}_{\ell+1}
\end{aligned}
\tag{S5}
$$

Hyperparameter search

We use algorithm S3 with 5-fold cross-validation to optimize hyperparameters for Random Forest, specifically n-estimators and max-depth, exploring the search space outlined in Table S3. Separate hyperparameter searches are conducted for the MLP head using ECFP and the MLP head using MolBERT features. Hyperparameters optimized for the MolBERT-based MLP head are also applied to the MLP head using VitroBERT features. Due to computational constraints, hyperparameter optimization is conducted with a single seed. Final model evaluations are repeated with 5 different seeds, and the mean and variance of the results are reported. We also compared three adaptation strategies for VitroBERT—full fine-tuning, Low-Rank Adaptation (LoRA), and linear probing—as shown in Table S5. For LoRA, we inserted rank-4 randomly initialized weights into the "query", "key", "value", and "dense" layers, keeping the original VitroBERT weights frozen. For linear probing, we trained a linear classifier on top of the frozen VitroBERT encoder. To ensure consistency, we used the same loss hyperparameters across all methods ($\alpha = 1$, $\gamma = 2$), which were previously optimized for VitroBERT with an MLP head. Fine-tuning was conducted for 5 epochs, while both linear probing and LoRA were trained for 110 epochs.

Table S3: We employ algorithm S3 with 5-fold cross validation to find the optimal hyperparameters by using this search space

Model family	Hyperparameter	Search space
Random Forest	N-estimators	$[2^n]$
	Maximum depth	$[1, 10] \cup [10 + 5n]$
MLP	Activation	[ReLU]
	Batch normalization	[True]
	Skip connection	[True]
	hidden layer dim	[128]
	Number of hidden layers	[1]
	α	$[0, 1]$
	γ	$[0, 1]$
	Dropout probability	[0.5]
	λ_2	$[10^{-5}, 10^{1}]$
	Optimizer	[Adam]
	Learning rate	$[10^{-3}]$
	Scheduler	[CosineAnnealingLR]
	T-max (LR cycle)	[10]
	Batch size	[64]
	Epochs	[410]

Results and Discussion

We initialized VitroBERT-invitro's encoder weights, PhysChemHead, and MaskingHead from a pretrained MolBERT model trained on 1.6 million SMILES strings for 100 epochs, with only the InvitroHead randomly initialized. The training curves (in Figure S5) at epoch 0 reflect MolBERT's baseline performance, and subsequent epochs demonstrate the model maintains performance in identifying masked tokens and physchem property modeling tasks while adapting to in vitro data.

For downstream evaluation, although AUPR serves as our primary evaluation metric due to the extreme class imbalance in our dataset, we also report ROC-AUC values (Table S4) for completeness and to facilitate comparison with previous studies.

Table S4: Evaluation of different models using ROC-AUC metrics across preclinical (Internal Hematology, Internal Pathology) and clinical (SIDER) domains.

Features	Predictive Head	In Vivo Loss	ROC-AUC			
			Hematology	Pathology	Clinical	Combined
ECFP	RF	-	0.81±0.026	0.63±0.027	0.61±0.012	0.65±0.008
	MLP	FL	0.80±0.042	0.65±0.044	0.62±0.023	0.66±0.010
MolBERT	MLP	FL	0.79±0.042	0.64±0.046	0.66±0.020	0.68±0.019
PhyschemBERT		FL^w	0.82±0.038	0.65±0.036	0.67±0.018	0.70±0.022
VitroBERT-internal		FL^w	0.86±0.035	0.70±0.044	0.67±0.014	0.71±0.018

Table S5: VitroBERT-Public, pretrained on public in vitro datasets, was evaluated on 8 biochemistry-related endpoints from TG-GATES. Performance metrics represent means across all tasks, with standard deviations calculated from 5 independent training runs using different random seeds. Evaluations were conducted using both random and structural similarity splits. Results demonstrate VitroBERT's superior performance over ChemBERTa, MolFormer, and MolBERT in both splitting strategies.

Features	Transfer Strategy	Random Split					Similarity Split				
		AUPR	ROC-AUC	Sens.	Spec.	MCC	AUPR	ROC-AUC	Sens.	Spec.	MCC
ChemBERTa-77M-MLM	MLP-head	0.41 ± 0.017	0.42 ± 0.036	0.41 ± 0.118	0.67 ± 0.118	0.16 ± 0.021	0.37 ± 0.016	0.51 ± 0.024	0.81 ± 0.053	0.51 ± 0.055	0.26 ± 0.030
ChemBERTa-77M-MTR	MLP-head	0.48 ± 0.025	0.55 ± 0.013	0.57 ± 0.070	0.61 ± 0.062	0.24 ± 0.022	0.32 ± 0.028	0.47 ± 0.023	0.72 ± 0.080	0.55 ± 0.098	0.22 ± 0.018
MoLFormer-XL-both-10pct	MLP-head	0.46 ± 0.045	0.48 ± 0.029	0.53 ± 0.175	0.60 ± 0.189	0.23 ± 0.022	0.40 ± 0.105	0.53 ± 0.085	0.72 ± 0.052	0.60 ± 0.135	0.28 ± 0.085
MolBERT	MLP-head	0.48 ± 0.015	0.62 ± 0.025	0.62 ± 0.025	0.65 ± 0.054	0.30 ± 0.024	0.37 ± 0.027	0.48 ± 0.022	0.64 ± 0.114	0.63 ± 0.111	0.25 ± 0.033
VitroBERT-public	LoRA	0.48 ± 0.033	0.52 ± 0.025	0.43 ± 0.051	0.77 ± 0.073	0.26 ± 0.034	0.39 ± 0.044	0.54 ± 0.049	0.71 ± 0.073	0.62 ± 0.050	0.29 ± 0.040
	Fine Tuning	0.44 ± 0.028	0.49 ± 0.056	0.57 ± 0.154	0.54 ± 0.118	0.20 ± 0.082	0.40 ± 0.072	0.53 ± 0.061	0.56 ± 0.115	0.78 ± 0.106	0.33 ± 0.078
	Linear probing	0.52 ± 0.015	0.63 ± 0.010	0.57 ± 0.017	0.72 ± 0.030	0.31 ± 0.021	0.41 ± 0.037	0.58 ± 0.008	0.76 ± 0.016	0.63 ± 0.029	0.34 ± 0.019
	MLP-head	0.62 ± 0.066	0.68 ± 0.031	0.61 ± 0.052	0.75 ± 0.078	0.37 ± 0.037	0.40 ± 0.037	0.55 ± 0.043	0.74 ± 0.162	0.64 ± 0.153	0.31 ± 0.027

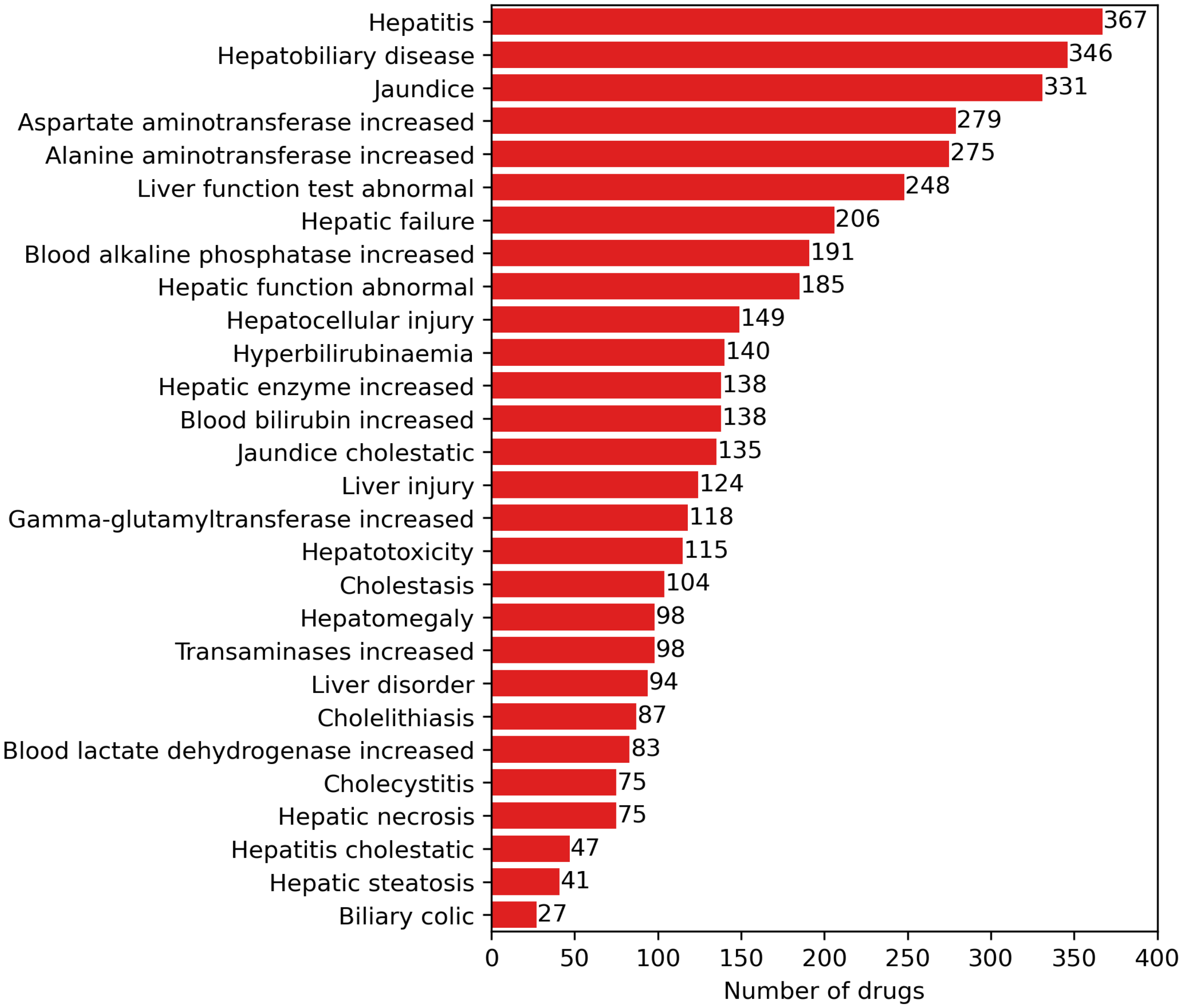

Figure S3: clinical: The distribution of drugs in 28 selected Hepatobiliary-related side effects(HepSEs)

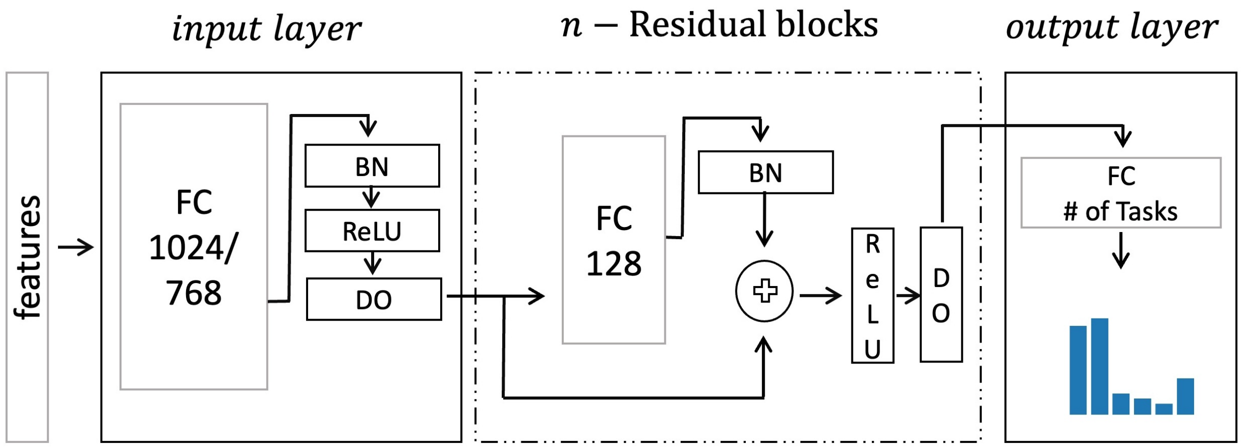

Figure S4: MLP head used for mapping ECFP and BERT features to preclinical and clinical toxicity estimations.

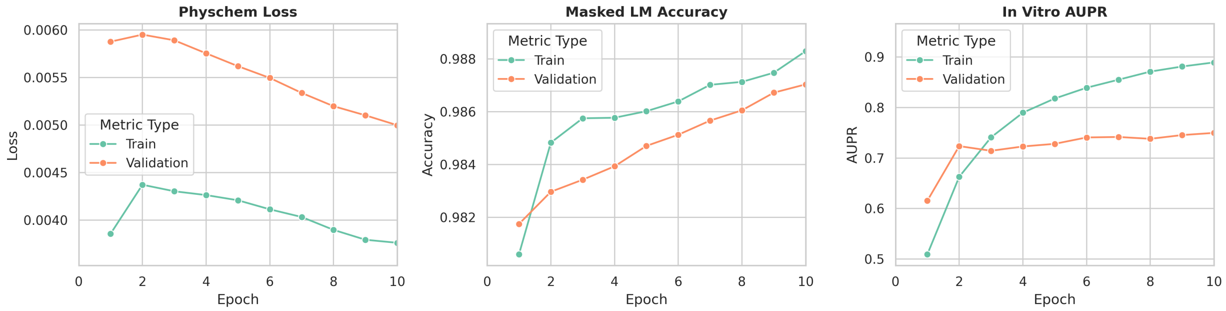

Figure S5: VitroBERT-public: Progression of Physchem Loss, Masked LM Accuracy, and in-vitro AUPR during pretraining stage.

Algorithm S1: Training procedure for VitroBERT variants with separate corrupted and clean sequence passes

Input:
$\text{MolBERT}_{\text{pretrained}}$, pretrained MolBERT model
$T_{\max}$, number of epochs
η_{encoder}, learning rate for encoder
η_{heads}, learning rate for heads
Output:
Trained VitroBERT variants
Data:
$\mathcal{D}_{\text{invitro\_train}}, \mathcal{D}_{\text{invitro\_val}}$, generated by Scaffold split

```
foreach VitroBERT variant do
    for t ← 1 to T_max do
        foreach B = {(s_i, s_i^corr, y_i^mask, y_i^physchem, y_i^invitro)}_{i=1}^{|B|} ⊂ D_invitro_train do

            h_corr ← Encoder(MolBERT_pretrained, s^corr) ;      // Corrupted sequence
            h_clean ← Encoder(MolBERT_pretrained, s) ;          // Clean sequence

            ŷ^mask ← MaskingHead(h_corr) ;                      // Head-specific estimation
            ŷ^physchem ← PhyschemHead(h_clean)
            ŷ^invitro ← InvitroHead(h_clean)

            L_mask ← ℒ_mask(ŷ^mask, y^mask) ;                   // Compute head-specific losses
            L_physchem ← ℒ_physchem(ŷ^physchem, y^physchem)
            L_invitro ← ℒ_invitro(ŷ^invitro, y^invitro)

            if Physchem-BERT then
                L_encoder ← L_mask + L_physchem ;                // Encoder loss
            end
            else if Vitro-BERT then
                L_encoder ← L_mask + L_physchem + L_invitro ;    // Encoder loss
            end

            θ_encoder ← θ_encoder − η_encoder ∇_{θ_encoder} L_encoder ;   // Update weights
            θ_mask ← θ_mask − η_heads ∇_{θ_mask} L_mask ;
            θ_physchem ← θ_physchem − η_heads ∇_{θ_physchem} L_physchem ;
            θ_invitro ← θ_invitro − η_heads ∇_{θ_invitro} L_invitro ;
        end
    end
end
return Trained VitroBERT variants
```

Algorithm S2: Genrerate ECFP or BERT features

```
Input:
    F_type, feature type (ECFP or BERT)
    D_invivo_SMILES, dataset of SMILES strings
    r_radius, radius for ECFP (only if F_type = ECFP)
    d_dim, embedding dimension for BERT (only if F_type = BERT)
Output:
    F, feature matrix (ECFP or BERT embeddings)
if F_type = ECFP then
    Initialize empty feature matrix F_ECFP ;
    foreach smiles in D_invivo_SMILES do
        mol ← MolFromSmiles(smiles) ;                // Convert to RDKit object
        fp ← GetMorganFingerprintAsBitVect(mol, r_radius) ;   // Compute ECFP
        Append fpto F_ECFP ;
    end
    F ← F_ECFP ;                                     // Return computed ECFP
else
    Initialize empty feature matrix F_BERT ;
    Load pretrained BERT model for molecular embeddings ;
    foreach smiles in D_invivo_SMILES do
        bert_input ← MolBERT_tokenizer(smiles) ;     // Generate tokens
        embedding ← BERT(bert_input) ;               // Extract BERT embedding
        Append embeddingto F_BERT ;
    end
    F ← F_BERT ;                                     // Return computed BERT embeddings
end
return F
```

Algorithm S3: Cross-validation and repeat-based testing procedure

Input:

K, number of folds

P_{set}, set of hyperparameters

R, number of repeats with different seeds

Data:

$\mathcal{D}_{\text{invivo\_train}}, \mathcal{D}_{\text{invivo\_test}}$, generated by Scaffold split (80/20 ratio)

Output:

$\mathcal{S}_{\text{val}}$, performance score of the best model ($f_{\hat{\phi}}$) on $\mathcal{D}_{\text{val}}$

$\mathcal{S}_{\text{test}}$, mean performance score of the best model ($f_{\hat{\phi}}$) on $\mathcal{D}_{\text{test}}$

$\mathcal{D}_{\text{invivo\_train}} = \{\mathcal{D}_1, \ldots, \mathcal{D}_K\}$, Random Stratified split $\mathcal{D}_{\text{invivo\_train}}$ into K subsets ;

foreach ϕ *in* P_{set} **do**

  for $k \leftarrow 1$ **to** K **do**

    $f_{\phi k}$, Train model on $\mathcal{D}_{-k}$ with hyperparameter ϕ ;

    $\mathcal{S}^{k}_{\text{val}}(\phi)$, Compute performance score of $f_{\phi k}$ on $\mathcal{D}_k$;

  end

  $\mathcal{S}_{\text{val}}(\phi) = \frac{1}{K}\sum_k \mathcal{S}^{k}_{\text{val}}(\phi)$; // k-fold mean score of f_ϕ

end

$\hat{\phi} \leftarrow \arg\max_{\phi} \mathcal{S}_{\text{val}}(\phi)$; // best hyperparameters

$\mathcal{S}_{\text{val}} \leftarrow \mathcal{S}_{\text{val}}(\hat{\phi})$; // best validation score

for $r \leftarrow 1$ **to** R **do**

  $f_{\hat{\phi},r}$, Train model on complete $\mathcal{D}_{\text{invivo\_train}}$ with hyperparameters $\hat{\phi}$ and seed r ;

  $\mathcal{S}^{(r)}_{\text{test}} \leftarrow$ Compute performance score of $f_{\hat{\phi},r}$ on $\mathcal{D}_{\text{invivo\_test}}$;

end

$\mathcal{S}_{\text{test}} \leftarrow \frac{1}{R}\sum_r \mathcal{S}^{(r)}_{\text{test}}$; // mean test score over R repeats

return $\mathcal{S}_{\text{val}}, \mathcal{S}_{\text{test}}$

Algorithm S4: The procedure for pre-training, feature extraction, and downstream training

Input:

F_{type}, feature type (ECFP or BERT)
$\mathcal{D}_{\text{invitro}}$, in vitro datasets for pretraining
T_{max}, number of epochs
η_{encoder}, learning rate for encoder
η_{heads}, learning rate for heads
K, number of folds for cross-validation
P_{set}, set of hyperparameters
R, number of repeats with different seeds
$\mathcal{D}_{\text{invivo}}$, downstream dataset
r_{radius}, radius for ECFP (if F_{type} = ECFP)
d_{dim}, embedding dimension for BERT (if F_{type} = BERT)

Output:

$\mathcal{S}_{\text{val}}$, validation performance score of the best model
$\mathcal{S}_{\text{test}}$, mean test performance score of the best model

Pre-training: ;
`Pretrain`($\mathcal{D}_{\text{invitro}}, T_{\text{max}}, \eta_{\text{encoder}}, \eta_{\text{heads}}$) ;

Feature Extraction: ;
$\mathbf{F} \leftarrow$ `ExtractFeatures`($F_{\text{type}}, \mathcal{D}_{\text{invivo}}, r_{\text{radius}}, d_{\text{dim}}$) ;

Cross-validation and Testing: ;
$\mathcal{S}_{\text{val}}, \mathcal{S}_{\text{test}} \leftarrow$ `CrossValidate`($K, P_{\text{set}}, R, \mathcal{D}_{\text{invivo}}$) ;
return $\mathcal{S}_{\text{val}}, \mathcal{S}_{\text{test}}$

References

(1) Igarashi, Y.; Nakatsu, N.; Yamashita, T.; Ono, A.; Ohno, Y.; Urushidani, T.; Yamada, H. Open TG-GATEs: a large-scale toxicogenomics database. *Nucleic Acids Research* **2015**, *43*, D921–D927.

(2) Moein, M.; Heinonen, M.; Mesens, N.; Chamanza, R.; Amuzie, C.; Will, Y.; Ceulemans, H.; Kaski, S.; Herman, D. Chemistry-Based Modeling on Phenotype-Based Drug-Induced Liver Injury Annotation: From Public to Proprietary Data. *Chemical Research in Toxicology* **2023**, *36*, 1238–1247.

(3) Kuhn, M.; Letunic, I.; Jensen, L. J.; Bork, P. The SIDER database of drugs and side effects. *Nucleic Acids Research* **2016**, *44*, D1075–D1079.

(4) Landrum, G. et al. rdkit/rdkit: 2021_09_4 (Q3 2021) Release. 2022; https://zenodo.org/record/5835217.

(5) Bemis, G. W.; Murcko, M. A. The Properties of Known Drugs. 1. Molecular Frameworks. *Journal of Medicinal Chemistry* **1996**, *39*, 2887–2893, Publisher: American Chemical Society.

(6) Pedregosa, F. et al. Scikit-learn: Machine Learning in Python. *Journal of Machine Learning Research* **2011**, *12*, 2825–2830.
